# Absence of Detectable Replication of Human Bocavirus Species 2 in Respiratory Tract

**DOI:** 10.3201/eid1509.090394

**Published:** 2009-09

**Authors:** Thaweesak Chieochansin, Amit Kapoor, Eric Delwart, Yong Poovorawan, Peter Simmonds

**Affiliations:** Chulalongkorn University and Hospital, Bangkok, Thailand (T. Chieochansin, Y. Poovorawan), University of California, San Francisco, California, USA (A. Kapoor, E. Delwart); University of Edinburgh, Edinburgh, Scotland, UK (P. Simmonds)

**Keywords:** Human bocavirus, parvovirus, PCR, respiratory infections, diagnosis, diarrhea, gastroenteritis, viruses, epidemiology, enteric diseases, dispatch

## Abstract

Human bocavirus (HBoV) commonly infects young children and is associated with respiratory disease; disease associations of the divergent HBoV-2 species are unknown. Frequent HBoV-2 detection in fecal samples indicated widespread circulation in the United Kingdom and Thailand, but its lack of detection among 6,524 respiratory samples indicates likely differences from HBoV-1 in tropism/pathogenesis.

Since its discovery in 2005 (*1*), human bocavirus (HBoV) has been the subject of intense investigation as a potential cause of human respiratory disease (*2*). In addition to respiratory tract and systemic infections, HBoV DNA sequences are frequently detected in fecal samples during primary infections (*3*,*4*), although a causative role in viral gastroenteritis has not been established (*5*–*7*). Other parvoviruses, including canine and bovine members of the genus *Bocavirus*, can replicate in the gastrointestinal tract and are often linked to enteric disease (*8*,*9*).

Until recently, published genetic analyses reported minimal sequence variability of HBoV strains; 2 genetic lineages differed in nucleotide sequence by only 2% in the virus protein 2 (VP2) gene (*10*). However, more divergent HBoV-like variants, provisionally designated as HBoV species 2 (HBoV-2), have been identified in fecal samples from children in Pakistan and the United Kingdom. These viruses show >20% nt sequence divergence (*11*). Published primer sequences for HBoV contain several mismatches with HBoV-2 sequences that may prevent their amplification (*11*). Thus, published surveys of HBoV prevalence likely report only HBoV-1. Therefore, HBoV-2 may represent an additional, currently undetected, agent in respiratory or enteric disease.

## The Study

To investigate HBoV-2, we developed new PCR-based detection methods for HBoV by using primer sets highly conserved between HBoV-1 and HBoV-2 and species-specific primers for HBoV-2. Large-scale screening of persons in the United Kingdom and Thailand was performed to compare virus detection frequencies in respiratory and fecal samples.

A total of 6,138 respiratory samples from 3,754 persons (2,018 male, 1,722 female, 14 sex unknown) during January 1, 2007–June 30, 2008, were obtained from the Specialist Virology Centre (Edinburgh, UK). Samples were not identified but epidemiologic and demographic information was retained (*12*,*13*). Samples comprised 3,065 nasopharyngeal swabs/aspirates (NPAs) and throat swabs (83%). A total of 386 NPAs were obtained from 386 persons (229 male, 154 female, 3 sex unknown) in Bangkok during February 16, 2006–July 20, 2008.

A total of 2,500 fecal samples were obtained from patients (1,093 male, 1,398 female, and 9 sex unknown) in Edinburgh predominantly with gastroenteritis or other enteric diseases referred for bacteriologic screening during March, June, and September 2008. A total of 530 fecal samples were obtained predominantly from children (179 boys and 138 girls) <5 years of age with diarrhea during July 12, 2007–July 25, 2008, and a control group without diarrhea (116 male, 96 female, 1 sex unknown) during March 4–December 2, 2007, in Bangkok.

DNA was extracted from 200-μL samples of pooled or individual specimens (respiratory samples, clarified fecal supernatant) into 40 μL Tris-EDTA buffer as described (*13*). Respiratory and fecal samples from Edinburgh were screened in pools of 10; both sample types from Bangkok were screened individually. Screening was performed by using nested primers conserved between HBoV-1 and HBoV-2 in the nucleoprotein (NP)–1 gene (universal primers: outer sense [position 2589 in DQ000496 st2 isolate (*1*)]: 5′-CCWATCGTCYTSYACTGCTTYGA-3′; outer antisense [2980]: 5′-TAGCYAAGTGTYTWBKGTACACATYAT-3′); inner sense [2727]: 5′-RTKSTGYGGBTTCTAYTGGCA-3′; and inner antisense [2963]: 5′-TACACATCATCCCARTAAYWACAT-3′).

Amplication conditions were 94°C for 2 min and 35 cycles at 94°C for 18 s, 50°C for 21 s, and 72°C for 1.5 min. Amplicons were differentiated by digestion with *Rsa*I. Fragments were sized by agarose gel electrophoresis. All known HBoV-1 sequences contain an *Rsa*I site between nt 2772 and nt 2773, resulting in fragments of 46 bp and 91 bp; this site is absent in HBoV-2 (undigested amplicon length of 237 bp).

Each pool or sample was additionally screened by using HBoV2-specific primers located in the nonstructural (NS)–1 gene (outer sense [1484]: 5′-AACAGATGGGCAAGCAGAAC-3′; outer antisense [2031]: 5′-AGGACAAAGGTCTCCAAGAGG-3′; inner sense [1618]: 5′-AACGATTGCAGACAACGCCTTATA-3′; and inner antisense [2019]: 5′-TCCAAGAGGAAATGAGTTTGG-3′; sites matching all known HBoV-2 variants and not matching HBoV-1 variants are underlined.) Amplification conditions were 95°C for 2 min; 5 cycles at 95°C for 45 s, 53°C for 1 min, and 72°C for min; and 35 cycles at 95°C for 30 s, 51°C for 30 s, and 72°C for 45 s. Positive pools of fecal samples from Edinburgh were divided and individual component samples were tested.

Respiratory and fecal samples from both centers were screened by using universal primers, and positive samples were digested with *Rsa*I. Undigested amplicons and some predicted HBoV-1 fragments (46 bp and 91 bp) were sequenced to confirm virus identity. All samples were additionally screened with HBoV-2–specific primers; 16 undigested samples were positive with HBoV-2–specific primers, and all samples identified as HBoV-1 were negative. Thus, species-specific primers enabled effective screening of HBoV-2 among samples with high frequencies of HBoV-1.

HBoV-positive fecal samples were generally restricted to children <5 years of age (25 from 30 infected children whose ages were known) (Figure, panel A; Table). Median age of children infected with HBoV-2 (7–12 months) was lower than that for those infected with HBoV-1 (1–2 years). Infections with HBoV-1 and HBoV-2 were observed at low frequencies in older persons (2 and 5 of 1,791 persons >35 years of age, respectively). For respiratory samples, HBoV-1 infections showed a similar peak incidence among children 1–2 years of age (Figure, panel B), similar to that observed for fecal samples. This age group was most frequently infected in our previous analyses of respiratory samples from Edinburgh (*12*). There were no differences in frequencies of HBoV-1 or HBoV-2 infection between male and female participants. Samples from Bangkok were divided into those from persons with diarrhea (327) and asymptomatic controls (213); detection of HBoV-1 and HBoV-2 was restricted to persons with diarrhea (n = 12 and 2, respectively).

**Figure:**
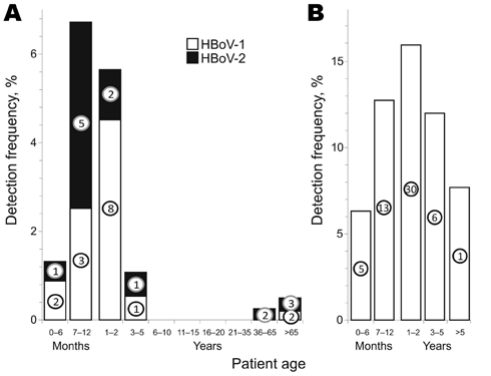
Age distribution of study participants with positive fecal (A) and respiratory (B) sample results for human bocavirus (HBoV), subdivided by HBoV species. Circles indicate numbers of positive samples in each category. Analysis of age distribution of persons with positive respiratory samples was restricted to samples from Bangkok, Thailand.

In contrast to its frequent detection in fecal samples, HBoV-2 was not detected in >6,500 respiratory samples (Table). However, high frequencies of HBoV-1 were recorded (14% among children in Bangkok and 3.4% among children in Edinburgh); the group from Edinburgh contained a substantial number of older children (37% >5 years of age).

**Table:** Frequency of human bocavirus species 1 and 2 in respiratory and fecal samples, United Kingdom and Thailand*

Sample type	Location	No. tested	Frequency, no. (%)	
			HBoV-1	HBoV-2
Fecal	Edinburgh, UK	2,500	6 (0.2)†	14 (0.6)†
	Bangkok, Thailand	530	10 (1.9)	2 (0.4)
Respiratory	Edinburgh, UK	6,138‡	67 (3.4)‡	0
	Bangkok, Thailand	386	55 (14.1)	0

*HBoV, human bocavirus. †Frequency among children <5 y of age: HBoV-1, 1.4%; HBoV-2, 3.1%. ‡Tested in 241 pools composed of 2,294 samples. Frequencies were determined by using the Poisson formula, *f* = ln(f_0_).

## Conclusions

Four conclusions can be drawn from this study. First, HBoV-2 circulates in 3 widely separated areas (United Kingdom, Thailand, and Pakistan [*11*]) and is likely distributed globally. Second, infections with HBoV-2 show a pattern of infecting young children, most <1 year of age. Third, absence of HBoV-2 in respiratory samples suggests a different tissue tropism that may influence its transmission route and ability to infect systemically and establish persistence. Determining the biologic basis for such differences will be useful in understanding the pathogenesis of HBoV-1–related respiratory disease. Fourth, at a practical level, absence of HBoV-2 in respiratory samples indicates no likely role for this virus in respiratory disease. Thus, screening methods may be adequate for detecting HBoV-associated respiratory disease. Nevertheless, the unexpectedly diverse human bocavirus group may contain additional variants with potential etiologic roles in respiratory or other diseases.

Since this study was completed, evidence for an interspecies HBoV-1/-2 recombinant associated with acute gastroenteritis has been obtained; the structural gene region was most closely related to HBoV-2, and NS1/NP-1 grouping with HBoV-1 (*14*). Although this recombinant would have been identified as HBoV-1 by using typing assays described in the current study, sequence analysis of HBoV-1–positive samples in this study and our previous study of respiratory samples from Edinburgh and Bangkok (*12*,*15*) identified only HBoV-1 in the study population, consistent with all other analyses of this sample type worldwide. Nevertheless, future typing assays should analyze both VP1/2 and NS/NP-1 to ensure that this and potentially other interspecies recombinants are identified. Investigation of genetic diversity of this group and development of effective screening methods for variants of HBoV is required for studies of human disease.
